# An Extended Two-Parameter Logistic Item Response Model to Handle Continuous Responses and Sparse Polytomous Responses

**DOI:** 10.1017/psy.2025.10044

**Published:** 2025-09-02

**Authors:** Seewoo Li, Hyo Jeong Shin

**Affiliations:** 1 Department of Education, University of California Los Angeles, Los Angeles, CA, USA; 2 Graduate School of Education, Sogang University, Seoul, South Korea

**Keywords:** computerized adaptive testing, continuous-bounded response, item response theory, sparse polytomous response, test linking and equating

## Abstract

The article proposes a novel item response theory model to handle continuous responses and sparse polytomous responses in psychological and educational measurement. The model extends the traditional two-parameter logistic model by incorporating a precision parameter, which, along with a beta distribution, forms an error component that accounts for the response continuity. Furthermore, transforming ordinal responses to a continuous scale enables the fitting of polytomous item responses while consistently applying three parameters per item for model parsimony. The model’s accuracy, stability, and computational efficiency in parameter estimation were examined. An empirical application demonstrated the model’s effectiveness in representing the characteristics of continuous item responses. Additionally, the model’s applicability to sparse polytomous data was supported by cross-validation results from another empirical dataset, which indicates that the model’s parsimony can enhance model-data fit compared to existing polytomous models.

## 1 Introduction

Item response theory (IRT) is being widely used in the field of psychology, education, and behavioral sciences, for many practical applications, such as data analysis, test equating and linking, developments of standard setting, and computerized adaptive testing (CAT). Numerous IRT models have been developed to take into account various features of item response data (van der Linden & Glas, 2010; van der Linden, 2016). In line with the development and expansion of IRT models, this article addresses two psychometric challenges.

Firstly, most of the IRT models used for test equating, standard setting, and CAT struggle with handling continuous response data. For example, in the age of generative artificial intelligence (AI), measuring skills, such as computer programming, often involves items that are continuously scored (e.g., Gerdes et al., 2010; Maiorana et al., 2015; Seo & Cho, 2018). Accurately accrediting test-takers on a reliable and comparable scale requires equating across different test dates, developing a standard setting, or constructing a proficiency scale. In these assessments, item responses can be completion rates, which are the ratio of completed subtasks to the total subtasks, where the number of subtasks often exceeds 10, or even 100. Moreover, continuous response formats, such as slider or visual analog scale (VAS) items, are increasingly used in computer-based assessments (e.g., Attali et al., 2022; García-Pérez, 2024; Gu, 2018; Open-Source Psychometrics Project, 2020; Toepoel & Funke, 2018; Vall-Llosera et al., 2020). Existing IRT models typically require discretizing continuous responses, leading to a loss of information. Instead, IRT models capable of directly handling continuous responses would provide more appropriate results for test equating and standard setting by preserving the continuous scale of the data.

Secondly, sparse item response data, characterized by a limited number of test-takers for each item or score category, is widely observed in the operational datasets (e.g., Casabianca et al., 2023; Jones et al., 2011; Kallinger et al., 2019; Mitchell et al., 2023). For example, ordinal response categories (e.g., 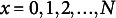 ) with few or no observations in certain categories hinder the application of polytomous IRT models, often necessitating post-hoc adjustments, such as collapsing score categories. This can be further exacerbated in the adaptive testing situation, when easy items dominate item banks to ensure test-taker engagement, resulting in sparse data in lower score categories, as most test-takers may get high scores on these items. Furthermore, a balanced incomplete block design (BIBD), which produces a sparse item response matrix, can be preferred to avoid the effect of test fatigue when many items are added to an item bank at once (e.g., Chen et al., 2023). This problem is expected to become more prevalent as item banks can be rapidly expanded through automated item generation (AIG) using large language models (LLMs) (Attali et al., 2022; Macat International, 2024; Shin et al., 2024; von Davier et al., 2024). Additionally, parsimonious IRT models can be beneficial for assessments with underrepresented groups, such as visually impaired students, where only a limited number of students participate in the assessment. In such cases, accurate and stable parameter estimation is threatened, but a parsimonious IRT model can be considered beneficial (Davey & Pitoniak, 2011; O’Neill et al., 2020).

Several IRT models have been proposed to deal with these challenges (see Section 2.3). However, they may not be suitable for operational applications of IRT in practice. Potential issues include assumption misalignment (Noel & Dauvier, 2007; Samejima, 1973), infeasible parameter estimation (Müller, 1987; Verhelst, 2019), and complex model interpretation (Müller, 1987; Samejima, 1973). Additionally, some models are based on factor analysis (FA) (Ferrando, 2001; Mellenbergh, 1994), and others focus on some special types of item responses (Chen et al., 2019; Kloft et al., 2023; Molenaar et al., 2022; Noel, 2014).

As a novel alternative approach, this article aims to propose a continuous-response IRT model: extended two-parameter logistic (E2PL) item response model, which can handle continuous item responses and sparse item responses. The E2PL extends the original two-parameter logistic model (2PL: Birnbaum, 1968) by incorporating an additional precision parameter that accounts for error, modeled using a beta distribution.

The proposed model offers several advantages. First, by benchmarking the generalized latent variable modeling framework (Skrondal & Rabe-Hesketh, 2004), although it does not strictly belong to the framework as it should be (see Section 3.3), its structure and interpretation are closely aligned with existing models, such as standard IRT and FA. For instance, indices analogous to communality and unique variance in FA can be easily derived, and parameters, such as factor loadings and intercepts, are explicitly specified. Consequently, the item parameters (i.e., item discrimination, difficulty, and precision) are straightforward to interpret. The discrimination and difficulty parameters retain interpretations similar to the 2PL model, while the precision parameter governs the error component. Specifically, the inverse of the precision parameter plays a role analogous to that of the dispersion parameter in the generalized linear model (GLM) framework (Ferrari & Cribari-Neto, 2004; McCullagh & Nelder, 1989; Skrondal & Rabe-Hesketh, 2004). Second, the error term’s beta distribution, which is the conjugate prior of binomial distribution, enables the model to accommodate a wide range of response distributions, including skewed or zero-one-inflated data. Third, it can effectively handle sparse score categories of polytomous item response data by transforming ordinal responses to a continuous scale (as demonstrated in Section 6). Being parsimonious, the E2PL can yield better model-data fit than conventional polytomous IRT models, especially when items have many score categories and sparse data limits the accuracy of parameter estimation. Lastly, the bell-shaped item information function of the E2PL is a useful feature that is expected to be used in adaptive testing to administer an item that provides maximum information.

The remainder of this article is organized as follows. Section 2 provides an overview of IRT and reviews existing IRT models for continuous responses. Section 3 presents the mathematical formulation of the E2PL with visual illustrations and through a comparison with the 2PL, discusses its differences from Noel & Dauvier (2007)’s model, and explicates its theoretical item response distribution. A simulation study in Section 4 evaluates the stability of the estimation algorithm, parameter recovery, and the computation time of parameter estimation. Sections 5 and 6 illustrate the application of the E2PL to empirical continuous response data and sparse polytomous data, respectively. Lastly, Section 7 discusses the E2PL’s potential and limitations and concludes the article. Detailed parameter estimation procedures are presented in the Appendix.

## 2 IRT and continuous item responses

### 2.1 IRT

Unlike classical test theory (CTT), which relies on summed scores, many IRT models use mathematical formulations that allow both item parameters (item characteristics) and ability parameters (test-takers’ latent proficiencies) to be calibrated and interpreted on a common scale. Furthermore, with appropriate scale conversions and test designs, scores from different tests can be adjusted and compared on a common scale, a process known as linking, equating, or vertical scaling, depending on the measurement context (Kolen, 2004). These features make IRT favorable for assessment developers and measurement practitioners in developing, administering, analyzing, and reporting tests. Additionally, assuming the item parameters in the item bank are accurate, IRT provides the foundational framework of adaptive testing, which enables test assembly, test selection, test stopping, and proficiency estimation.

#### 2.1.1 2PL model

**Figure 1:**
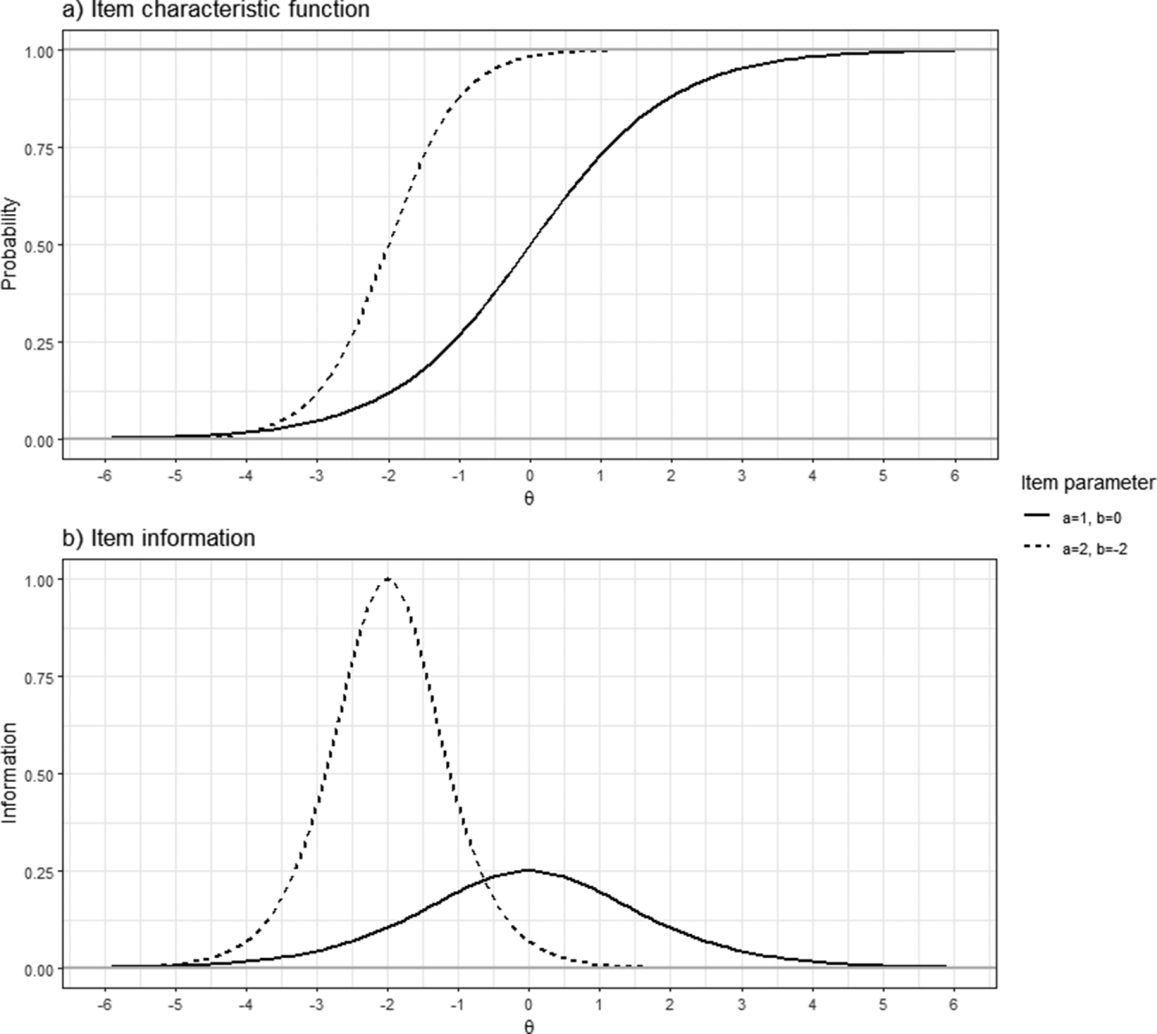
ICFs and item information functions of the 2PL. *Note:* For illustrative purposes, values of item parameters are set to 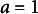 and 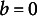 for the solid lines and to 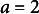 and 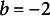 for the dotted lines.

As one of the most popular IRT models and for its direct connection to the E2PL, we briefly review the 2PL (Birnbaum, 1968). Assuming one dichotomous item response 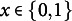 from a single test-taker for brevity, the probability of a correct response ( 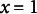 ) can be expressed as follows: (1)

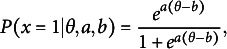

where 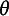 , *a*, and *b* are ability parameter, item discrimination parameter, and item difficulty parameter, respectively. Depending on varying 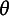 values, the probability of a correct response ranges from 0 to 1, exhibiting an *S*-shaped curve as in Panel (a) of Figure 1. The item difficulty parameter *b* determines the inflection point of the symmetric curve at which the probability becomes 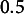 , and the item discrimination parameter *a* determines the steepness of the curve.

##### Scale transformation

If *a*, *b*, and 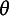 in Equation (1) are replaced with 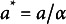 , 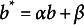 , and 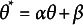 , it is always satisfied that 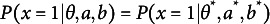 for arbitrary 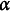 and 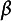 . Using this linear transformation, test linking, equating, and vertical scaling can be achieved in a more flexible and interpretable way.

##### Information function

Supposing 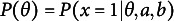 , the item information function 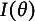 can be written as follows: (2)

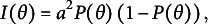

which is Panel (b) of Figure 1. The item information function has its highest value at 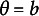 , and the peak of the function gets higher with a larger *a* value. The function tapers to 0 as 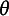 diverges to positive or negative infinity. The bell-shape of the function implies that the amount of information is concentrated around 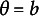 . In particular, the item information function provides useful insight about the range of targeted test-takers’ ability levels during the assessment design and CAT.

##### Asymptotic standard error

Since the asymptotic standard error of the ability parameter estimate 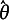 is 
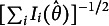
 , where *i* denotes the item, the amount of information directly affects the accuracy of the ability parameter estimate.

### 2.2 Modeling continuous item responses

Along with the advancement of IRT to address a variety of measurement challenges, the introduction of diverse item formats and assessment designs has further driven the development of models capable of handling various types of item responses. These include, but are not limited to, dichotomous, polytomous, and continuous responses.

While numerous IRT models have been developed to address dichotomous and polytomous data, relatively few models have been proposed to flexibly accommodate continuous item responses. One reason for this lag is the traditional compartmentalization of latent variable modeling, which generally categorizes IRT as a framework for analyzing discrete observed variables alongside continuous latent variables (Bartholomew et al., 2011). According to this classification, continuous observed variables (i.e., continuous item responses) are typically handled using FA. Although this distinction is not a strict rule (Cai, 2012), it is often presented in introductory texts on latent variable modeling for its conceptual simplicity. This convention may have led researchers to either apply FA or discrete-response IRT models to continuous-response data.

Another factor contributing to the slower development of continuous-response IRT models is the historical reliance on paper-based assessments, where items are predominantly scored dichotomously or polytomously. It is only more recently, with advancements in information and computer technologies, that continuous item formats have gained broader use in practice (e.g., Attali et al., 2022; García-Pérez, 2024; Gu, 2018; Open-Source Psychometrics Project, 2020; Toepoel & Funke, 2018).

### 2.3 Existing models for continuous item responses

This section reviews existing IRT models for continuous item responses. Each model has its own distinct characteristics and purposes, but they may present limitations for test development, linking, or equating due to their parameterization, underlying assumptions, or structural features. Certain models are only briefly mentioned here, as they are less relevant to the scope of this article: Mellenbergh (1994) and Ferrando (2001) adopted the identity link function in modeling continuous response, a typical choice within the FA framework, Noel (2014) proposed a model for unfolding responses using the Dirichlet distribution, Chen et al. (2019)’s model assumes a bounded latent space, and Kloft et al. (2023) focused on modeling interval responses on a continuous range using the Dirichlet distribution.

- Samejima (1973)’s model: By taking the number of categories in the graded response model (Samejima, 1969) to infinity, the model postulates an ability parameter and three item parameters: item discrimination, difficulty, and scaling. When a 2PL-type parameterization is applied, the modified difficulty parameter is a combination of the scaling parameter and the original difficulty parameter. The model is based on the normality assumption on the logit-transformed response, and item information is constant across the latent 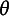 scale.
- Müller (1987)’s model: Similar to Samejima (1973)’s model, the model is an extension of Andrich (1982)’s rating scale model by taking the number of categories to infinity. The parameters of interest are ability, item difficulty, and item dispersion. As a Rasch-type model, it holds the specific objectivity property, enabling conditional maximum likelihood estimation. However, parameter estimation may not be practically feasible (Verhelst, 2019) and the absence of the item discrimination parameter can present limitations in practical applications. In addition, item responses are projected on the latent scale using a uniform distribution.
- Noel & Dauvier (2007)’s model: The model utilizes the beta distribution in modeling continuous bounded response, and has ability, item difficulty, and item dispersion parameters. In contrast to the other models that project item responses on the latent space, the model directly handles item responses on their original domain using the beta distribution. The model assumes that the responses are generated from an interpolation mechanism. This model has the most relevant feature to the E2PL, thus, their relationships are discussed in Section 3.4.
- Verhelst (2019)’s model: The model is a direct extension of the Rasch model (Rasch, 1960), having only ability parameter and item difficulty parameter. Despite its simplicity, the model postulates neither probabilistic distribution nor an additional parameter to account for the continuity of responses, such as the scaling or dispersion parameter in the other models discussed above. Furthermore, parameter estimation may not be practically feasible (Verhelst, 2019), and the effectiveness of the practical application has not been examined.
- Molenaar et al. (2022)’s model: Under the assumption that 0s and 1s are inflated, 0s and 1s are separated from the other responses through mixture modeling. Then, responses between 0 and 1 ( 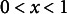 ) can be modeled with any type of model listed above. However, considering that seemingly zero-one-inflated data can be properly modeled by a beta distribution, it is challenging to tell whether 0s and 1s are truly inflated.

Building on the review of existing methods, the practical need for a new model can be formally motivated as follows. Suppose we are designing an assessment that includes both dichotomous and continuous items, using the 2PL model for dichotomous items to capture item discrimination and difficulty. This structure can be observed in practice, for instance, in programming assessments that combine multiple-choice questions (scored dichotomously) with coding tasks evaluated as percentage-correct (continuous). In this context, the zero-one inflated model by Molenaar et al. (2022) is not suitable for general measurement purposes. Additionally, the models proposed by Müller (1987), Verhelst (2019), and Noel & Dauvier (2007) do not incorporate item discrimination parameters, making them incompatible with the 2PL framework. The interpolation mechanism assumed in Noel & Dauvier (2007) is also unlikely to be appropriate for item responses such as percentage-correct scores. Finally, while Samejima (1973)’s model includes discrimination and difficulty parameters, they are not aligned with those of the 2PL model. In particular, its expected value is given by 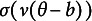 (Wang & Zeng, 1998), where 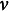 is a scaling parameter and 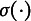 denotes the sigmoid function, whereas the 2PL uses 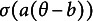 as in Equation (1), with *a* representing item discrimination. Therefore, a new model that is compatible with the 2PL, as well as capable of handling continuous item responses, is required.

## 3 E2PL model

### 3.1 Formulation of the E2PL

#### 3.1.1 Model equations

Moving from the 2PL (see Equation (1)) to the E2PL, we make a transition from binary to continuous response. Below, the response *x* takes a real number ( 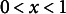 ), thus, the modeled value is no longer a probability for the Bernoulli distribution but an observable quantity. The model equations can be written as follows: (3)

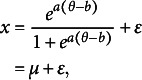


(4)

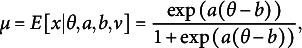


(5)

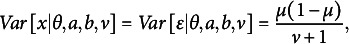

and (6)

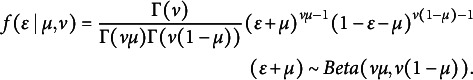

The expected response 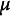 is equal to the model equation of the 2PL (Equation (1)). Particularly, the error term 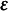 is introduced to account for the continuous nature of the response using a beta distribution. Through a comparison with the 2PL, Section 3.2.1 illustrates how this error term reflects the continuity. The error term follows a shifted beta distribution where the amount of the shift is 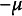 ( 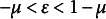 ). The precision parameter 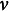 can represent the degree to which the density is concentrated around 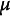 . Using Equations (3) and (6), the model can be rewritten in a probabilistic form: (7)

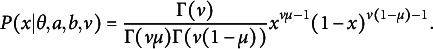



#### 3.1.2 Information function

The item information function of the E2PL can be expressed using trigamma function 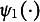 : (8)

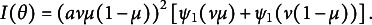

In general, the item information function resembles a bell-shaped curve, peaking at 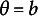 . However, its shape can vary depending on the precision parameter 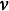 . When 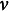 is close to 3, the function can take a *W*-shaped form, whereas for values of 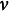 less than 2, it tends to look like a bell-shaped curve flipped upside down. Notably, even with small values of 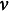 , the item information in the E2PL model is greater than that of the 2PL model, assuming the same *a* and *b* parameters are used for both. The greater information of the E2PL can be attributed to its more refined response structure compared with binary responses. Unlike many other dichotomous or polytomous models, the information function in the E2PL does not approach zero as 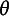 approaches 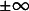 , instead asymptotically approaching 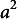 from below.

#### 3.1.3 Likelihood

We can add subscripts to the equations to express a likelihood function of data. Letting 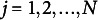 denote test takers and 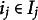 be an *i*th item among 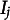 items that the *j*th test taker responded to, the likelihood can be expressed as follows: (9)

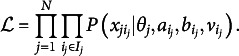

The individual item response probabilities (Equation (7)) are multiplied under the assumptions that responses within individuals are independent conditional on the latent trait level 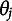 (i.e., the local independence assumption) and that responses are statistically independent across individuals.

#### 3.1.4 Model-data fit and standardized residuals

Following the approach proposed by Ferrari & Cribari-Neto (2004) in the context of beta regression, the standardized residual for test taker *j* and item *i* can be computed as: (10)

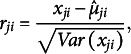

where 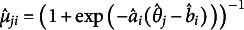 and 
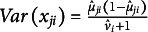
 are from Equations (4) and (5). Residual analysis based on this formulation can provide evidence of model misspecification.

The overall model-data fit can be assessed using a pseudo 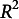 , defined as the squared correlation between the log-odds of 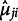 and the log-odds of 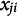 (Ferrari & Cribari-Neto, 2004). Additionally, *K*-fold cross-validation provides a more robust estimate of predictive performance. In this case, the log-likelihood under the beta distribution or the root mean squared error (RMSE) of the standardized residuals (i.e., 
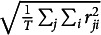
 ) can serve as a loss function, where *T* is the total number of item responses evaluated.

Accordingly, for the current version of the E2PL, we advocate the use of standardized residuals, pseudo 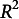 , and *K*-fold cross-validation for evaluating model fit, due to their interpretability. More advanced diagnostic and fit assessment methods (e.g., Espinheira et al., 2019; Espinheira et al., 2008; Smithson & Verkuilen, 2006) may be appropriate for future extensions of E2PL.

#### 3.1.5 Parameter estimation

Item parameters can be estimated through marginal maximum likelihood using the expectation–maximization algorithm (MML-EM: Bock & Aitkin, 1981), where the marginal likelihood is obtained by integrating the likelihood 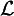 with respect to 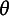 . Conventionally, the 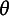 distribution is assumed to follow the standard normal distribution. Several types of scores can be used as ability parameter estimates, such as expected *a posteriori* (EAP), maximum likelihood estimate (MLE), or weighted likelihood estimate (WLE). Details of the parameter estimation are provided in the Appendix.

### 3.2 Item characteristic function (ICF)

#### 3.2.1 Comparison with the 2PL

Within the generalized latent variable modeling framework (Skrondal & Rabe-Hesketh, 2004), both FA and IRT models can be expressed in the form 
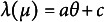
 , where 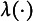 denotes the link function, typically the identity link for FA and the logit link for IRT. In this formulation, the slope parameter *a* corresponds to the factor loading, and *c* represents the intercept. Specifically, in the 2PL model, *a* is interpreted as item discrimination, while item difficulty is given by 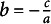 . The E2PL model adopts this same structural formulation, allowing for analogous interpretations of the *a* and *b* parameters. However, the key distinction lies in the modeling of the dispersion structure: the 2PL model assumes a Bernoulli distribution for the observed responses, whereas the E2PL employs a beta distribution. This difference in distributional assumption differentiates the two models.

**Figure 2:**
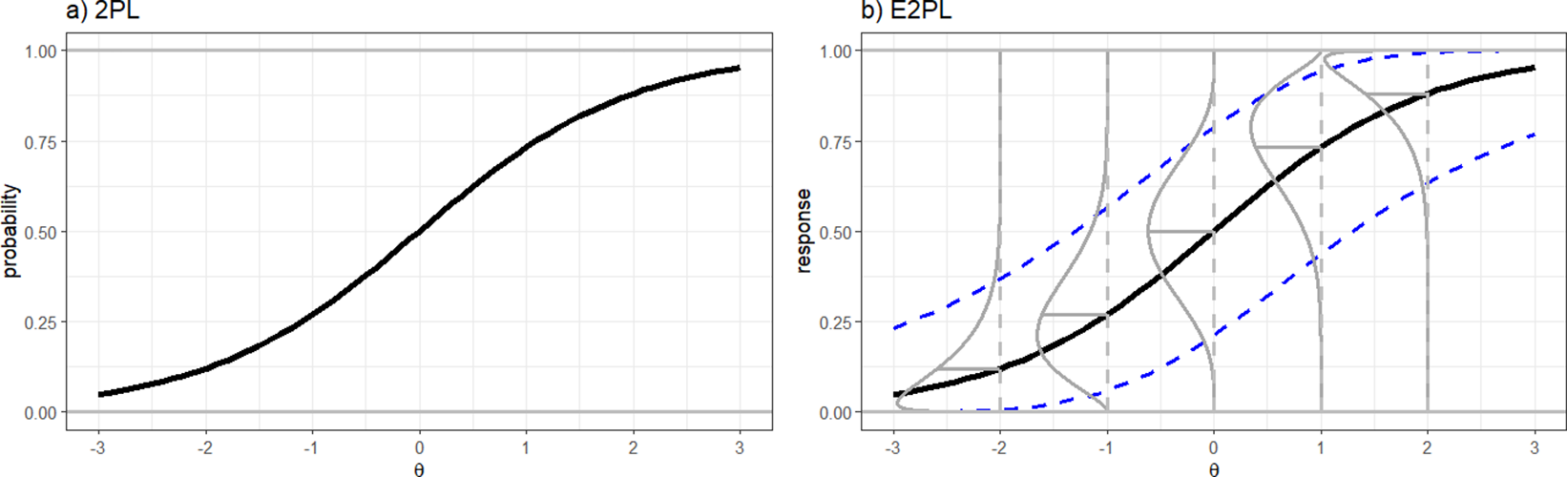
ICFs of the 2PL and the E2PL. *Note:* For the illustration, values of item parameters are set to 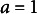 and 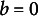 for both functions and 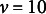 for the E2PL. The *y*-axis is probability in Panel (a) and response in Panel (b). In Panel (b), the 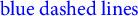 indicate 95% interval conditional on 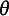 . The 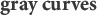 are probability densities of continuous item responses for the selected 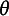 values of -2, -1, 0, 1, and 2. The 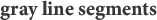 on the 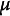 -curve indicate the means of the densities.

Figure 2 visually illustrates similarities and differences between the 2PL and the E2PL. Due to their common item discrimination and difficulty parameters, the ICF of the 2PL in Panel (a) and the 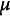 curve of the E2PL in Panel (b) are identical. Thus, item interpretations of the two models would be similar or, for convenience, interchangeable. In addition, an identical linear transformation can be carried out for the two models for test linking or equating. Therefore, for mixed-format data of dichotomous and continuous item responses, adopting the pair of the 2PL and the E2PL simplifies the model interpretation and the scale transformation.

Compared with the 2PL’s ICF, the ICF of the E2PL in Panel (b) of Figure 2 includes the blue and gray curves to display the beta distribution, the error component of the E2PL. In line with the change of the *y*-axis from 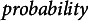 to 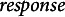 , the error term of the E2PL accounts for the continuous scale of the response. The distribution (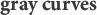) and the 95% interval (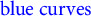) illustrate how the error distribution changes according to 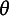 .

#### 3.2.2 Role of the item parameters

**Figure 3:**
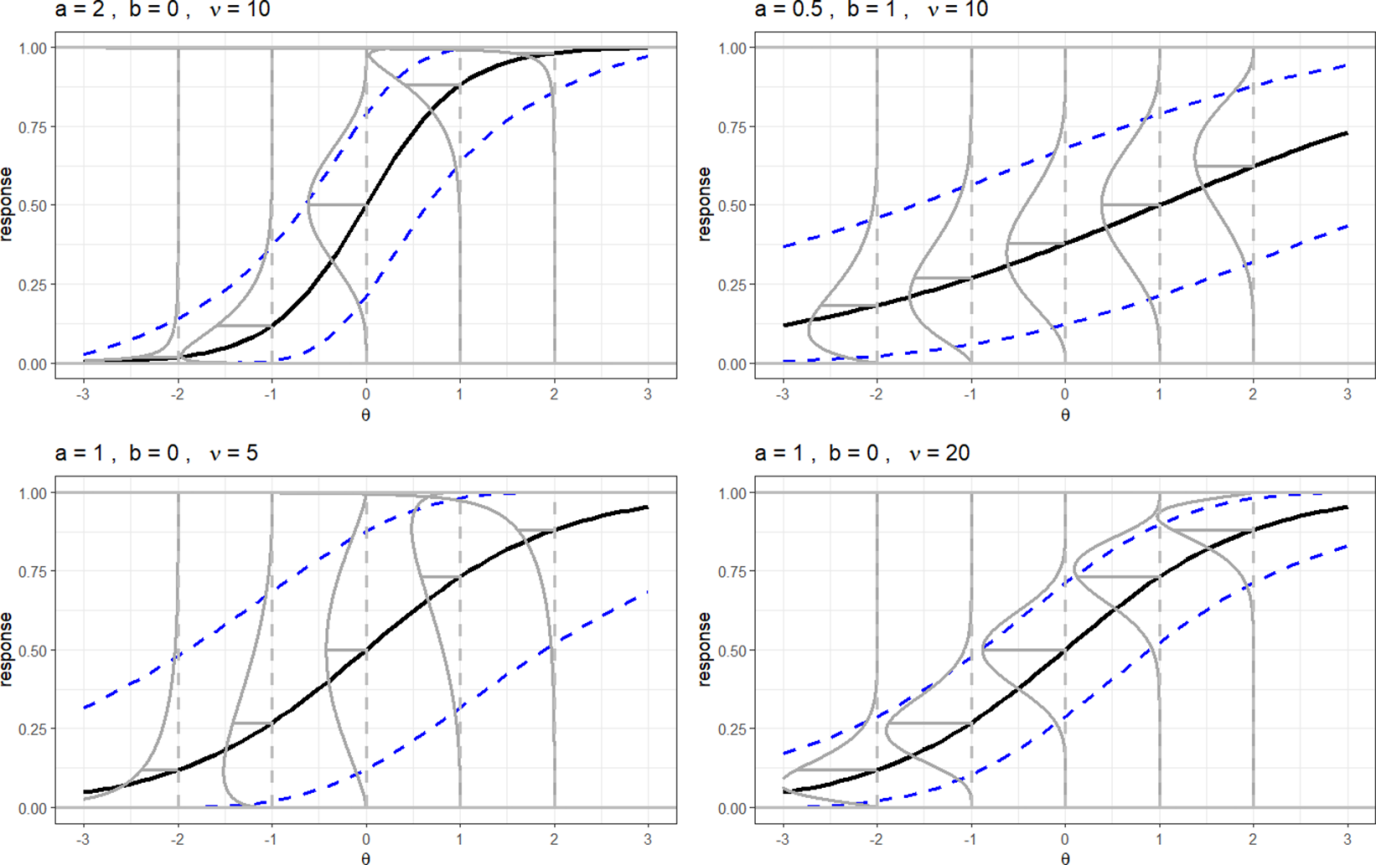
ICFs of the E2PL with varying parameter values. *Note:* The 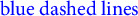 indicate 95% interval conditional on 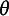 . The 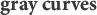 are probability densities of continuous item responses for selected 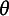 values of -2, -1, 0, 1, and 2. The 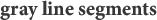 on the 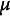 -curve indicate the means of the densities.

Figure 3 visually illustrates how item parameters influence ICFs in the E2PL. The upper left and right panels show that the effects of the *a* and *b* parameters on ICFs are structurally identical to those in the 2PL model: the *a* parameter alters the steepness of ICFs and the *b* parameter shifts ICFs along the 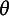 -axis. However, unlike the 2PL, where the *Y*-axis represents probability, the E2PL models the response directly on a continuous scale. Accordingly, the *a* parameter determines the slope of the expected response (i.e., the 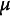 curve), and the expected response reaches 0.5 when 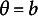 .

Focusing now on the role of the precision parameter 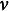 , when the expected response curves (**solid black lines**) are held constant, the error distributions (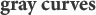) are also identical across panels with the same 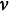 . For example, in the upper-left panel at 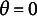 and the upper-right panel at 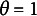 , the distributions are identical because both yield an expected response of 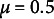 under the same precision level 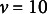 .

To illustrate the effect of the precision parameter, the precision parameter 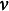 is varied between the lower left and right panels. It can be seen that the precision parameter modified only the error variances (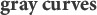) and the width of the interval (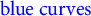). In brief, after the mean curve (**solid black curves**) is determined by the *a* and *b* parameters, the precision parameter 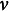 determines the dispersion, or concentration, of ICFs.

### 3.3 Communality and unique dispersion

The formulation of the E2PL is well-aligned with the generalized latent variable modeling framework (Skrondal & Rabe-Hesketh, 2004), as it models the mean structure 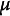 using the logit link function and introduces the precision parameter 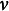 to handle the dispersion of data. Here, we use the term *dispersion* instead of *variance* to indicate the role of 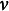 , since statistical variance of the beta distribution depends on 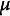 as in Equation (5). Meanwhile, the beta distribution does not provide a statistically independent structure between 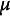 and 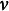 (Ferrari & Cribari-Neto, 2004), which differentiates the E2PL from the generalized latent variable modeling framework (McCullagh & Nelder, 1989; Skrondal & Rabe-Hesketh, 2004). However, this is a desirable property that allows the model to account for the effects of the item parameters during the calculation of the residual dispersion represented by 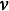 .

To mathematically illustrate the aforementioned points, let 
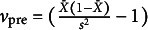
 be the degree of precision for an item before fitting the E2PL with sample mean 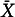 and sample variance 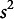 , and 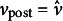 be the estimate of the precision parameter of the item in Equations (3)–(7). The subscripts for the items are omitted for notational brevity. Then, using the FA notation from MacCallum (2009), 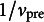 and 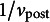 are analogous to the sample variance 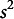 and the estimate of the unique variance 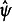 in FA, respectively. As a result, the proportion of the unique dispersion of this item becomes 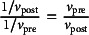 , and the communality 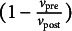 indicates the proportion of the uncertainty explained by the latent variable. The relationships above are also applicable to multidimensional 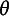 .

**Figure 4:**
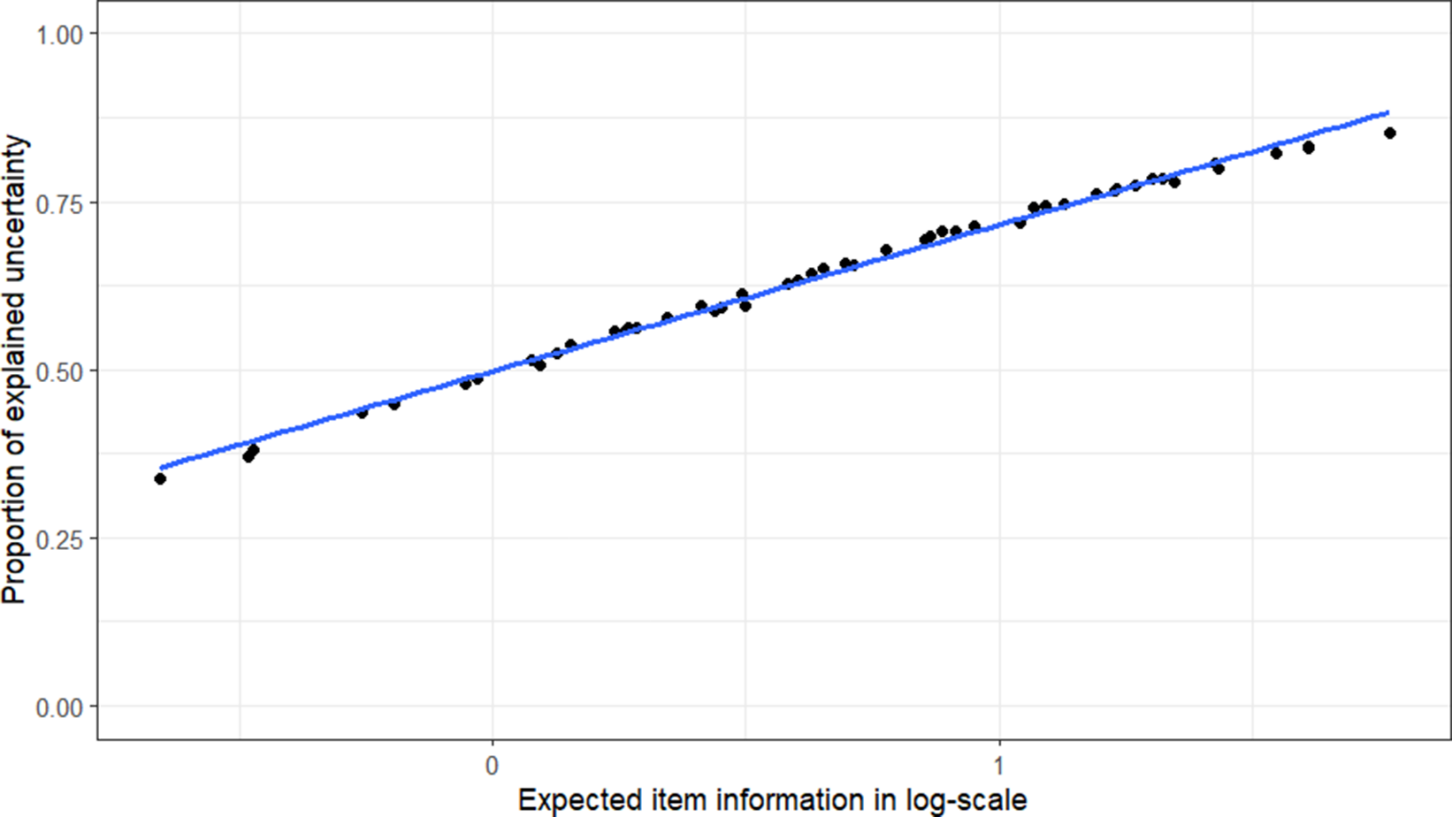
The relationships between the proportion of explained uncertainty 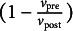 and expected item information 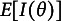 . *Note:* The expected item information values are calculated as 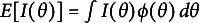 , where 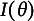 is the item information function in Equation (8) and the standard normal distribution 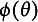 is used as the latent distribution. The figure is obtained from a randomly generated data for 100,000 test takers and 50 items, where 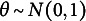 , 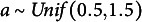 , 
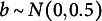
 , and 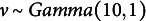 .

Figure 4 shows an almost linear relationship between the explained dispersion and the expected item information in log scale. This trend well reflects the conventional practice in IRT to take item parameters into account, rather than excluding them when explaining the uncertainty of the data. Notably, the formulation of the communality and unique dispersion, as well as their relationships to the item information, are not subject to the scale transformation in Section 2.1.1, as the transformation does not affect 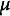 .

### 3.4 Differences between the E2PL and Noel and Dauvier’s model

It would be worthwhile to clarify the differences between the E2PL and Noel & Dauvier (2007)’s model as they are closely related to one another. Although they introduced a Rasch-type model without item discrimination parameter, it can be easily added to the model. The following discussion assumes Noel and Dauvier’s model with the inclusion of the item discrimination parameter to make a fair comparison.

Item responses of Noel and Dauvier’s model are assumed to be a manifestation of the interpolation mechanism; an interpolation of one weight ( 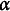 ) pulling a response toward 0 and another weight ( 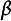 ) pulling it toward 1 (i.e., 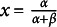 ), thereby adopting the shape–shape parameterization of the beta distribution. In other words, they modeled the response using 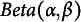 , where the expected value of the response *x* is the interpolation of the two parameters (i.e., 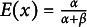 ). In comparison, without assuming a particular mechanism on item responses, the E2PL adopts the mean–precision parameterization, which is a widely used practice in beta regression (Ferrari & Cribari-Neto, 2004).

While the conditional means of the two models (i.e., the 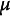 terms) can be identically expressed using the *a* and *b* parameters, the two models differ in their treatment of precision. In the E2PL model, the conditional variance—after accounting for 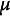 —is solely governed by the precision parameter 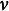 , enabling a clear separation of the error component as shown in Equation (3). In contrast, the conditional variance in Noel and Dauvier’s model remains a function of all item parameters even after conditioning on 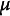 . Following the parameterization of Ferrari & Cribari-Neto (2004), the precision parameter in Noel’s model is given by 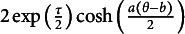 , where 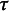 is an additional parameter to account for dispersion. In the E2PL model, by contrast, the precision is directly represented by 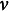 . Because the precision term in Noel and Dauvier’s model depends on the latent scale, scale transformations and the derivation of quantities, such as communality and unique dispersion, require additional consideration.

### 3.5 Distribution of item responses

Each IRT model assumes a particular distribution for item responses. For instance, when the 2PL’s item parameters are 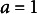 and 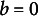 and 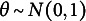 , the expected probability of observing an item response of 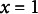 is 0.5.

Similarly, the item response distributions of the E2PL can be derived when the latent distribution is specified. Assuming the standard normal distribution on the latent variable, the response distributions can be mathematically expressed as follows: (11)

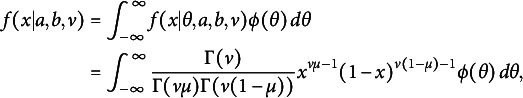

where the 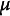 is defined in Equation (4) and 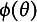 denotes the standard normal latent distribution. The distribution depends only on item parameters after integrating out the latent variable 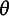 .

**Figure 5:**
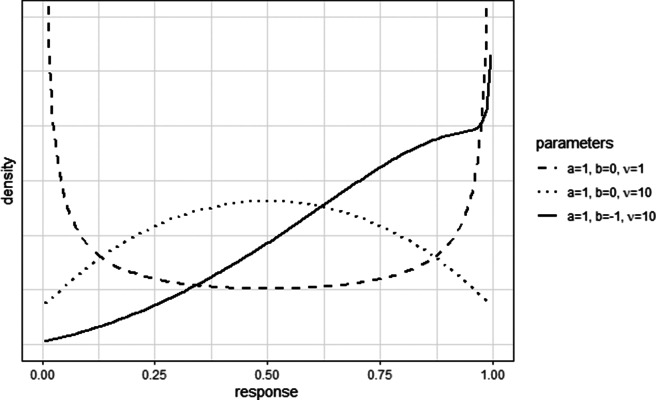
Response distributions of the E2PL. *Note:* The distributions are derived from the standard normal latent distribution. The distributions are numerically approximated.

Figure 5 shows that skewed or zero-one-inflated response distributions can be generated by the model, as well as bell-shaped distributions. Firstly, when the difficulty parameter *b* is different from the mean of the latent distribution, a skewed distribution can be formulated. For instance, the mass of the solid line’s density ( 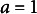 , 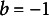 , 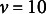 ) is more concentrated near 1 than 0, as the item is relatively easy for the population: 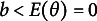 . In comparison, the densities of the dotted and dashed lines ( 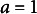 , 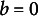 , 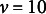 ; 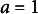 , 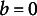 , 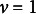 ) are both symmetric as their difficulty parameters are equal to the population mean.

Secondly, zero-one-inflated response distributions can be formulated with a small value of 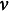 . For example, the dashed line ( 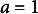 , 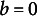 , 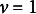 ) shows that both 0 and 1 are inflated even when the difficulty parameter *b* is equal to the population mean. In a strict sense, it may not be an actual zero-one-inflated distribution as the domain of the beta distribution does not contain 0 and 1. However, in practice, responses are almost always rounded to a certain point (e.g., to the nearest hundredth), producing 0s and 1s. Additionally, skewed zero-one-inflated response distributions can be formulated when the precision parameter is small and 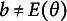 .

### 3.6 Preprocessing item responses

To apply the E2PL, a simple transformation of item responses is often necessary, when raw data include minimum and maximum values (e.g., 0% and 100%) that fall outside of the domain of the beta distribution, which excludes 0 and 1. For example, item responses collected as percentages typically include these values as minimum and maximum scores.

**Figure 6:**
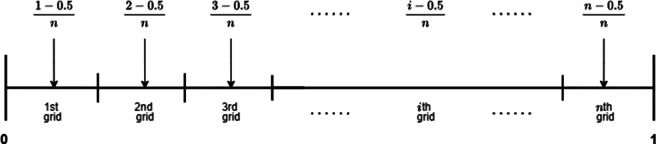
Mapping raw scores on a unit interval.

To adjust raw responses to fit within the beta distribution while preserving their key characteristics, we recommend the preprocessing scheme shown in Figure 6. This approach divides the open interval (0, 1) into equally spaced grids based on the smallest unit of observation in the raw data and maps the raw responses to the midpoints of these grids. For instance, if percentage responses are measured in 1% increments, the raw scores from 0% to 100% are mapped to the midpoints of 101 equally spaced grids: 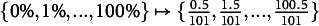 . Figure 6 visually illustrates this method.

This transformation is useful in cases where values approach the absolute maximum or minimum, allowing for meaningful representation of such scores. In practical contexts, discrepancies often exist between mathematical maximums, defined as the highest possible score, and practical maximums, which may exhibit slight variations even at the upper boundary. For instance, some maximum scores represent model solutions that are qualitatively distinct from those that merely achieve the highest possible score. A similar rationale applies to minimum scores, such as the differences between zero scores due to blank responses and other minimum scores. This approach supports using the open interval of the beta distribution, rather than the closed interval, to better reflect the nuanced characteristics of item responses near the extremes. Additionally, other transformations, such as slightly adjusting the minimum and maximum values (Noel & Dauvier, 2007), could also be applied when they more accurately capture the characteristics of item responses.

Furthermore, this transformation is justifiable when each item comprises multiple subtasks (see Sections 5 and 6). In such cases, a more sophisticated modeling approach, such as the testlet response model (Bradlow et al., 1999; Wainer et al., 2007), is often appropriate for accounting for local item dependencies. However, testlet models can be sensitive to sample size constraints, potentially increasing estimation error due to the bias–variance trade-off and threatening the validity of inferences. While more parsimonious models may be preferable under limited sample conditions, standard polytomous IRT models are often not suitable alternatives, as their response categories typically do not align directly with individual subtasks. Thus, the proposed transformation provides a practical alternative to testlet modeling by enabling the use of the E2PL that maintains fidelity to the item structure while avoiding issues associated with small samples.

## 4 Simulation study

A simulation study is conducted to assess the recovery of item parameters and the stability of the parameter estimates of the E2PL. Data were generated and the model was fitted using the IRTest package in R (Li, 2025), with evaluation metrics computed using built-in R functions (R Core Team, 2024). The simulation code is publicly available at https://github.com/SeewooLi/E2PL_simulation_study.

### 4.1 Data generation and model-fitting

The simulation study utilizes a set of 12 items, designed as the factorial combination of the following item parameters: discrimination ( 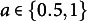 ), difficulty ( 
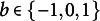
 ), and precision ( 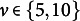 ). Sample sizes of 250, 500, and 1000 are used, and for each sample size, 200 sets of item response data are generated based on the specified item parameters.

**Table 1:** Biases and RMSEs in parameter recovery

	Sample	Parameter			Bias (RMSE)		
Item	size	*a*	*b*	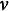	*a*	*b*	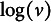
1		0.5	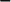 1	5	0.01 (0.07)	0.01 (0.18)	0.02 (0.09)
2		0.5	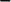 1	10	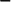 0.00 (0.05)	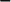 0.01 (0.15)	0.00 (0.10)
3		0.5	0	5	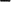 0.01 (0.06)	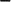 0.01 (0.12)	0.00 (0.08)
4		0.5	0	10	0.00 (0.05)	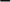 0.01 (0.10)	0.01 (0.09)
5		0.5	1	5	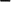 0.00 (0.06)	0.02 (0.18)	0.01 (0.10)
6	250	0.5	1	10	0.00 (0.05)	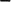 0.00 (0.14)	0.01 (0.09)
7		1	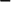 1	5	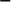 0.01 (0.08)	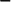 0.02 (0.12)	0.02 (0.10)
8		1	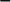 1	10	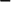 0.00 (0.08)	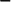 0.01 (0.11)	0.01 (0.11)
9		1	0	5	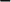 0.01 (0.08)	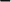 0.00 (0.09)	0.02 (0.10)
10		1	0	10	0.00 (0.06)	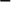 0.00 (0.08)	0.02 (0.10)
11		1	1	5	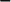 0.02 (0.08)	0.01 (0.11)	0.01 (0.10)
12		1	1	10	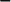 0.00 (0.07)	0.00 (0.09)	0.02 (0.10)
1		0.5	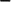 1	5	0.01 (0.05)	0.00 (0.13)	0.00 (0.06)
2		0.5	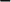 1	10	0.00 (0.03)	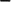 0.00 (0.10)	0.00 (0.06)
3		0.5	0	5	0.00 (0.04)	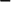 0.00 (0.08)	0.00 (0.06)
4		0.5	0	10	0.01 (0.03)	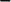 0.01 (0.06)	0.01 (0.07)
5		0.5	1	5	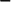 0.00 (0.04)	0.01 (0.11)	0.01 (0.06)
6	500	0.5	1	10	0.00 (0.04)	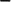 0.01 (0.10)	0.01 (0.06)
7		1	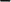 1	5	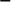 0.03 (0.06)	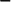 0.03 (0.08)	0.00 (0.07)
8		1	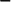 1	10	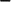 0.00 (0.05)	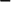 0.01 (0.06)	0.02 (0.08)
9		1	0	5	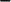 0.01 (0.06)	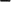 0.00 (0.05)	0.01 (0.07)
10		1	0	10	0.00 (0.05)	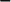 0.01 (0.05)	0.01 (0.08)
11		1	1	5	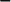 0.03 (0.06)	0.02 (0.08)	0.00 (0.07)
12		1	1	10	0.00 (0.04)	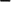 0.01 (0.06)	0.01 (0.09)
1		0.5	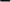 1	5	0.00 (0.03)	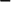 0.00 (0.09)	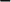 0.00 (0.04)
2		0.5	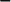 1	10	0.00 (0.03)	0.00 (0.07)	0.00 (0.04)
3		0.5	0	5	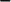 0.00 (0.03)	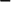 0.00 (0.06)	0.01 (0.04)
4		0.5	0	10	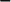 0.00 (0.02)	0.00 (0.05)	0.00 (0.04)
5		0.5	1	5	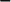 0.00 (0.03)	0.01 (0.09)	0.00 (0.04)
6	1000	0.5	1	10	0.00 (0.03)	0.00 (0.07)	0.00 (0.04)
7		1	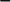 1	5	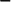 0.02 (0.04)	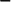 0.02 (0.06)	0.00 (0.05)
8		1	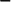 1	10	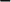 0.00 (0.03)	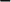 0.01 (0.05)	0.01 (0.05)
9		1	0	5	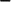 0.00 (0.04)	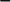 0.00 (0.04)	0.00 (0.05)
10		1	0	10	0.00 (0.03)	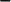 0.00 (0.03)	0.01 (0.05)
11		1	1	5	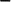 0.02 (0.04)	0.01 (0.06)	0.01 (0.05)
12		1	1	10	0.00 (0.03)	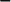 0.01 (0.05)	0.01 (0.05)

Model fitting is conducted using the IRTest package (Li, 2025). A custom function IRTest_ Cont, which is developed for this study, implements the MML-EM procedure (Bock & Aitkin, 1981). Convergence of the MML-EM procedure is defined as the point at which the maximum change in parameter estimates falls below 0.0001 within a maximum of 200 EM iterations, ensuring robust model fitting in this simulation. To address the challenge of directly estimating a bounded parameter (i.e., 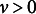 ), 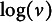 is estimated and the changes in 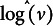 are tracked. The MML-EM procedure employs 121 equally spaced quadrature points ranging from 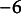 to 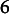 , assuming the standard normal latent distribution. EAP scores are used to estimate the ability parameter.

### 4.2 Evaluation criteria

The accuracy of parameter recovery is assessed using bias and RMSE for the item parameter estimates across 200 replications. To evaluate computational efficiency, mean computation time (MCT) is calculated. An AMD Ryzen 7 5700G processor is used for the study. Finally, the stability of the estimation process is confirmed by verifying the convergence of the estimation procedures throughout the simulation study.

### 4.3 Results

All 600 MML-EM procedures (200 replications 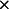 3 sample sizes) successfully converged within 200 iterations, demonstrating robust stability in the estimation process.

Table 1 summarizes the biases and RMSEs for parameter recovery. The estimates for all three item parameters (discrimination, difficulty, and precision) were nearly unbiased, with the largest observed bias being less than 0.03. Notably, parameter recovery was satisfactory even for the smallest sample size of 250, and the estimation of the precision parameter remained accurate and stable across conditions.

An additional simulation was conducted to examine parameter recovery for items with small *a* or 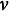 values. Two more items were added to the simulation design: Item 13 with 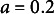 , 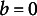 , and 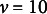 , and Item 14 with 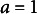 , 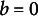 , and 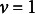 . For Item 13, 95% of the item responses fall within the interval 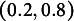 , whereas for Item 14, two-thirds of the responses fall outside this interval. The parameter estimates for Item 13 are unbiased with comparatively larger RMSE for 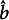 , which decreased from 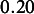 to 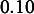 as the sample size increased from 250 to 1000. In contrast, the discrimination parameter *a* for Item 14 exhibited a negative bias of approximately 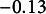 across the three sample sizes, which can be attributed to the rounding of item responses to the nearest value on a discretized scale ranging from 0.00005 to 0.99995 in steps of 0.0001. This suggests that more fine-grained response options are necessary when the precision parameter 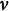 is small to account for the areas near 0 and 1.

The MCT for convergence is shown in Table 2. On average, the MML-EM procedure took 8.74, 14.50, and 26.29 seconds to converge for sample sizes of 250, 500, and 1000, respectively. These times are considered efficient given the data size, a convergence threshold of 0.0001, and the 121 quadrature points used. The RMSE( 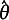 ) is included for reference; as expected, with test length held constant, RMSE( 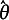 ) values were consistent across the simulation conditions.

**Table 2:** MCT and RMSE( 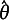 )

Sample size	MCT	RMSE( 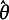 )
250	8.74	0.27
500	14.50	0.27
1000	26.29	0.27

*Note:* The MCTs are measured in seconds.

## 5 Empirical Illustration 1: Continuous response data

### 5.1 Data

To illustrate the application and interpretation of the E2PL model, we use empirical data from a company located in South Korea. This company developed and administered an assessment to measure the programming skills of its employees, aiming to establish a reliable and valid item bank through psychometric methods.

For this analysis, we use 12 items, each containing one to four tasks, with each task subdivided into 4 to 60 subtasks depending on the item’s purpose. For instance, Item 3 comprises two tasks, with 50 subtasks in the first task and 60 subtasks in the second. The data is unbalanced, with responses from 1,732 participants who answered between one and ten items. Most participants responded to either one item ( 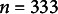 ) or two items ( 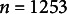 ), and only one participant responded to ten items.

Following the preprocessing procedure outlined in Section 3.6, the binary subtask scores were aggregated and mapped to a unit interval, transforming them into continuous item responses. First, a task-level score is computed. For example, if a participant completes 17 subtasks out of 50 (resulting in the 18th category from 0 to 50), the task-level score is calculated as 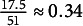 . The item score is then the average of the task-level scores, producing a continuous value that represents the percentage of task completion.

As in Section 4, the IRTest (Li, 2025) package is utilized for the model fitting. Given the sparsity of the data, a more lenient convergence threshold of 0.01 is adopted. To evaluate the overall model-data fit, the pseudo 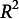 (see Section 3.1.4) was calculated after model estimation, resulting in a value of 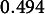 .

**Figure 7:**
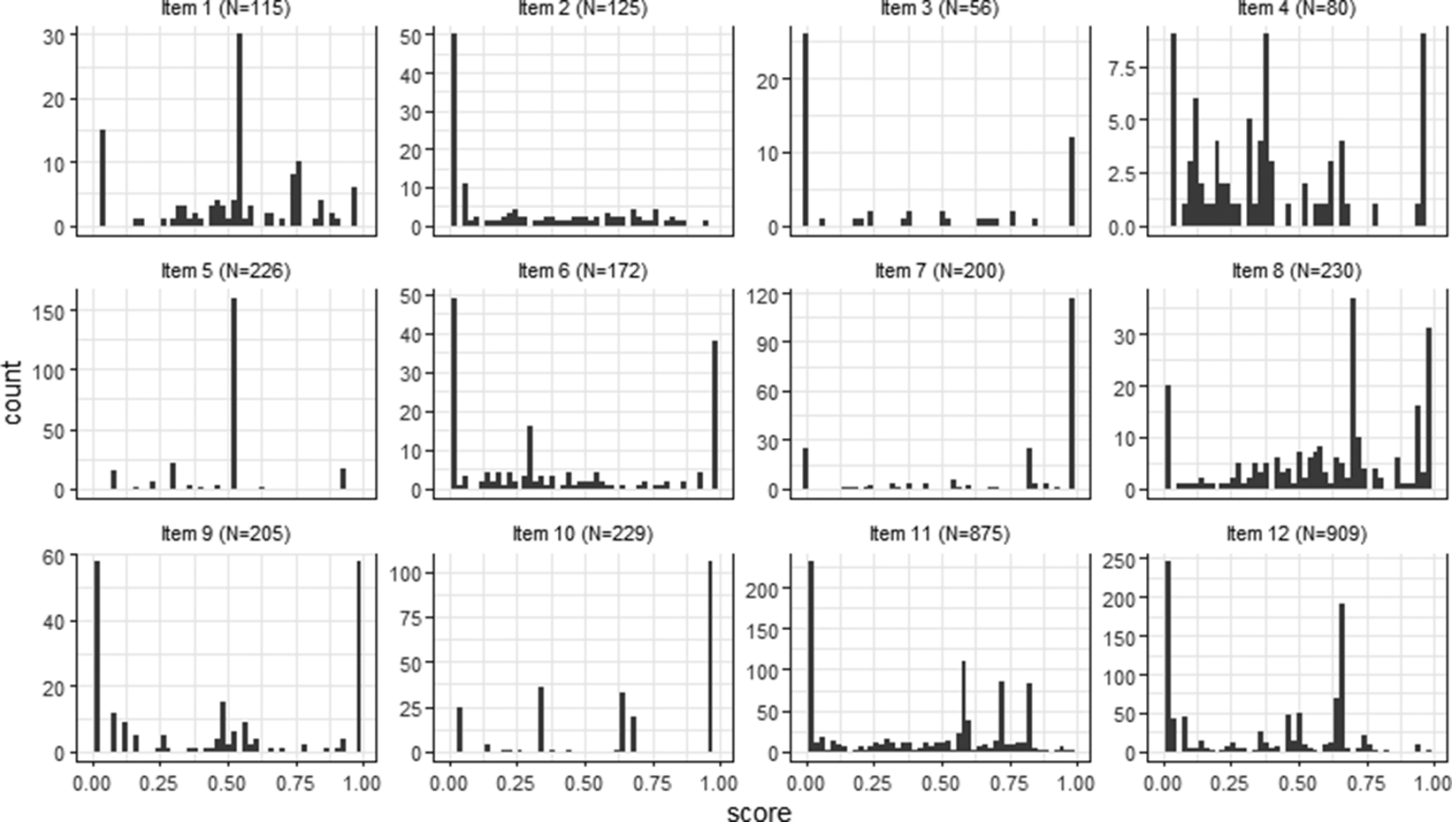
Item response distributions of the programming assessment dataset.

Figure 7 presents histograms of the item responses, which reflect the continuous scores obtained from the mapping process in Section 3.6. Across all items, the unit interval is densely populated with item responses. In an extreme case, for example, Item 11 yields 172 unique response values, highlighting the challenges of handling such data with a polytomous IRT model.

**Table 3:** Item parameter estimates and communalities of the programming assessment dataset

Item	*a*	*b*	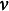	Communality
1	0.79	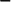 0.03	5.03	0.43
2	0.15	6.85	2.15	0.31
3	0.40	1.07	0.76	0.48
4	0.35	1.02	2.14	0.13
5	0.48	0.17	8.87	0.19
6	0.48	0.47	1.01	0.33
7	0.21	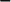 3.65	0.84	0.46
8	1.46	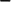 0.32	9.53	0.81
9	0.65	0.17	1.20	0.50
10	0.58	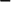 1.00	1.98	0.43
11	0.67	0.75	2.22	0.32
12	0.59	1.17	2.62	0.30

Except for Items 1, 5, and 8, most item response distributions are skewed or exhibit zero-one inflation. For example, Item 2 responses are concentrated near 0, while Item 9 shows inflation near both 0 and 1. Note that the 0s and 1s mentioned above are not exact 0s and 1s, but the closest value to 0 and 1, respectively.

### 5.2 Item parameter estimates and ICFs

**Figure 8:**
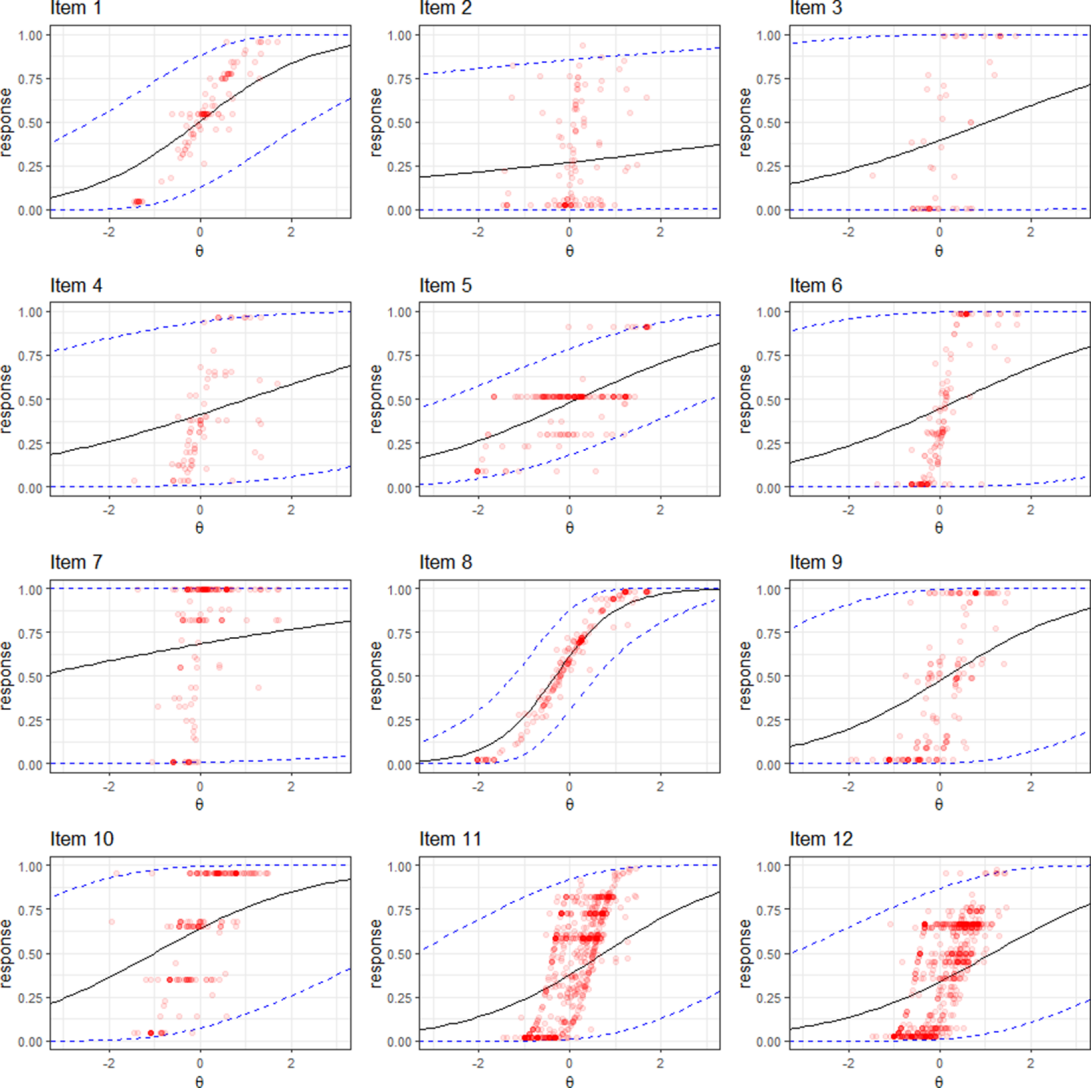
ICFs and item responses of the programming assessment dataset. *Note:* The **black lines** are the expected response 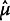 , the 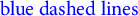 indicate the 95% confidence interval, and the 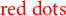 are the observed responses.

The item parameter estimates are shown in Table 3, and Figure 8 displays the ICFs alongside individual item responses. Figure 8 demonstrates that the E2PL model provides a good fit for the observed item responses. Notably, when the precision parameter 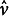 is relatively high (e.g., Item 8), responses are tightly clustered around the 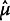 line. Conversely, for items with lower precision parameters (e.g., Items 3, 6, and 7), responses conditional on 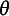 are more dispersed across the response range. For instance, in the interval 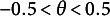 for Item 7, responses span nearly the entire range from 0 to 1.

Given that the estimated 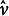 values for Items 3, 6, and 7 are near or below 1, these items could potentially be simplified into dichotomous items to facilitate scoring. However, any such modifications should not be based solely on the 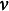 parameter, as scoring decisions are also influenced by the overall test design and item format.

### 5.3 Item information functions

**Figure 9:**
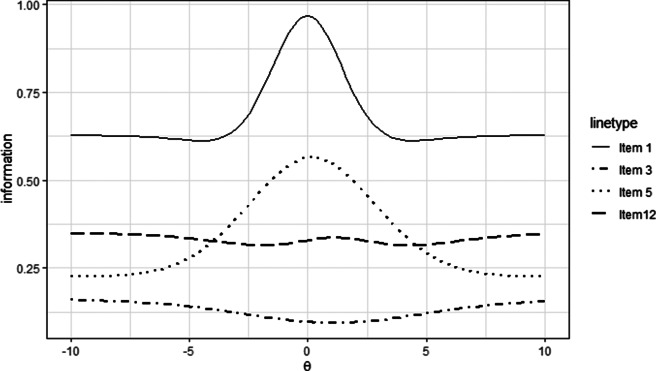
Item information functions of the programming assessment dataset. *Note:* Items 1, 3, 5, and 12 are selected for illustrative purposes.

Referring to Equation (8), the magnitude of the item information functions in the E2PL model depends on both *a* and 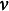 . For illustrative purposes, the item information functions of Items 1, 3, 5, and 12 are shown in Figure 9. The functions are drawn on a wide range of 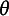 -axis to capture the overall shapes. The information functions for Items 1 and 5 are bell-shaped due to their large 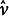 values. Each function reaches its peak at the corresponding estimated item difficulty parameter 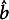 (Item 1 at 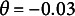 and Item 5 at 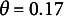 ).

In contrast, due to their small 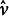 values, the item information functions for Items 3 and 12 exhibit local maxima at their respective estimated difficulty parameters 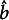 (Item 3 at 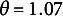 and Item 12 at 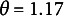 ). As 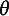 approaches 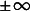 , all of the information functions asymptotically approach 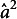 from below.

### 5.4 Discussion

The E2PL model enables the analysis of continuous item response data without requiring discretization, which allows for the extraction of more information compared to discrete data. Specifically, the 2PL has a trade-off between the coverage of the latent space and the amount of item information (see Equation (2)). A high discrimination parameter results in a concentrated area of high item information (a sharp peak in the item information function that quickly diminishes), whereas a low discrimination parameter spreads item information more evenly across a broader latent range, though with a lower overall peak.

In contrast, the E2PL delivers high item information while covering a larger portion of the latent space. For instance, Item 5, with a precision parameter estimate of 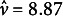 , maintains high item information, and its relatively low discrimination parameter ( 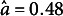 ) allows the item information to span almost the entire latent continuum of interest (i.e., 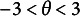 ).

Furthermore, as an extension of the 2PL, the parameter interpretation of the E2PL remains straightforward. The discrimination and difficulty parameters share the same mathematical interpretation as in the 2PL. The newly introduced precision parameter represents the concentration of item responses around the *S*-shaped mean curve, independent of the latent 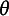 scale. In addition, the scale transformation for the 2PL illustrated in Section 2.1.1 is directly applicable to the E2PL parameter estimates presented in this section, maintaining consistency with the 2PL framework.

Lastly, the communality indices presented in Table 3 reflect the proportion of uncertainty in each item, represented by 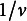 , accounted for by the latent variable. The relatively low communality values for some items (e.g., Items 4 and 5) may indicate potential model misfit, such as multidimensionality in the latent construct or limitations in the design of items intended to assess programming skills.

## 6 Empirical Illustration 2: Sparse polytomous data

With the growing interest in online learning and increased accessibility to digital devices, more psychometric data are being collected through online platforms. At the same time, controlling item exposure remains a critical concern in the development of computer-based assessments. As a result, adaptive testing is gaining more attention, generally requiring large quantities of items (e.g., Chen et al., 2023; Yan et al., 2016). In such testing situations, item parameters are generally treated as fixed (i.e., pre-calibrated) based on pilot tests. However, during these pilot tests, sparse item response data are often collected, particularly when items are scored polytomously.

If an IRT model is used to analyze the sparse polytomous data, it may pose challenges to the parameter estimation (Davey & Pitoniak, 2011; O’Neill et al., 2020). Moreover, the problem would get worse if the items have many score categories, as the number of item parameters in polytomous IRT models tends to increase with the number of categories (Noel, 2014). In addition to continuous response data, analyses of sparse polytomous data can be enhanced by leveraging the E2PL, which may provide a robust framework for such situations.

### 6.1 Data

A sparse polytomous dataset has been retrieved from an assessment developed by Macat (Macat International, 2024) for assessing students’ critical thinking skills. According to the assessment design, students are assigned different sets of 24 items. Every item has four statements that are scored as either correct or incorrect, with the total score for an item being the sum of correct responses ( 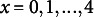 ). For this analysis, data from 3,502 participants and 94 items were utilized. Originally, there were 96 items, but two misbehaving items were excluded.

The item response matrix, with dimensions of 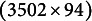 , contains 82,173 valid responses, which constitute approximately 25% of the total possible responses, with the remainder being missing data. This sparseness arises because each student responded to only 24 items. As a result, the number of responses per item varies, ranging from 593 to 1,210. Moreover, among the 470 score categories across the 94 items (94 items 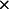 5 categories), responses were observed fewer than ten times for 35 categories, and fewer than 30 times for 112 categories. For Item 11, the fourth category ( 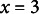 ) had no observations.

Following the preprocessing procedure outlined in Section 3.6, the item responses were transformed to a unit interval. This transformation divides the unit interval into five equally spaced sections, each with a width of 0.2. The midpoints of these sections were assigned as the continuous item responses for each category: 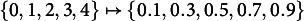 . These converted responses were then treated as continuous item responses in the E2PL.

### 6.2 Comparison of E2PL and GPCM

#### 6.2.1 Visual comparison

**Figure 10:**
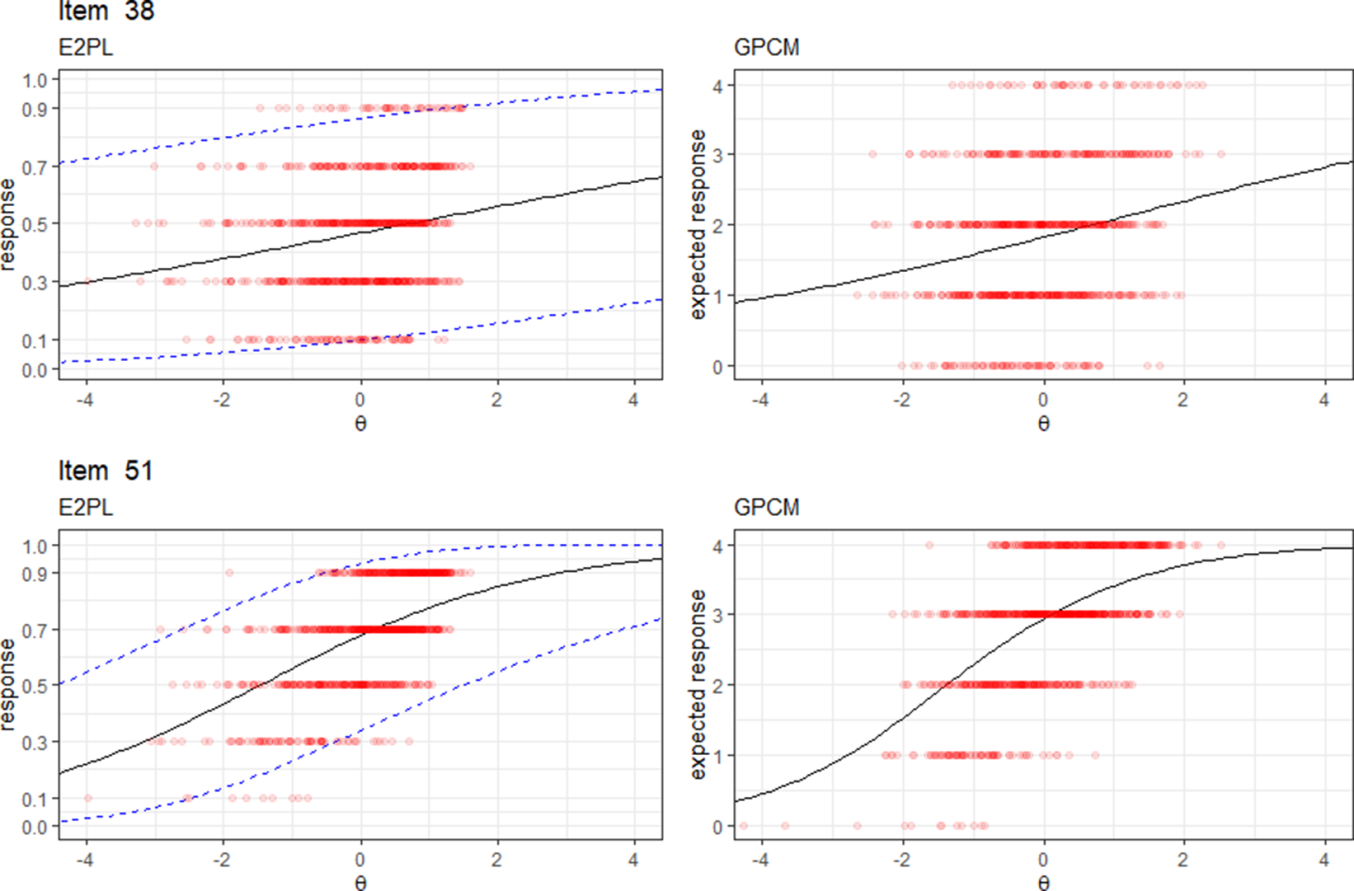
Visual comparison between the E2PL and GPCM for the selected items. *Note:* Items 38 and 51 are selected for illustrative purposes.

The comparison between the two models begins with a visual examination of their ICFs, as shown in Figure 10. For illustrative purposes, two items (Item 38 and Item 51) out of the 94 total items are selected. The figure displays the mean curve and 95% interval of the E2PL alongside the expected response curve of the generalized partial credit model (GPCM; Muraki, 1992). Additionally, individual item responses are plotted against the ICFs, providing a visual assessment of how each model represents item responses. Based on how the item responses align with the ICFs, the two models demonstrate comparable performance, which supports the use of the E2PL for sparse polytomous data.

#### 6.2.2 Quantitative comparison

Next, the model-data fit of the E2PL is quantitatively compared to the GPCM using *K*-fold cross-validation. The unit of prediction for this comparison is the item response. For both models, raw scores ( 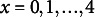 ) are predicted. The E2PL, which generates predictions on the unit interval, transforms these into the original scale ( 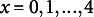 ), which is the inverse process of the one described in Figure 6. Given the size of the dataset, a 10-fold cross-validation approach is employed. On each iteration of the 10-fold cross-validation, RMSE is computed to assess the predictive performance of both models. RMSE is calculated on the raw score scale to avoid favoring either model.

RMSEs from the two models are listed in Table 4. RMSEs of the E2PL were consistently lower than those of the GPCM. Overall, the average RMSE of the E2PL was 0.92 and that of the GPCM was 1.00, indicating that the E2PL had a better predictive performance than the GPCM. Additionally, as a measure of the overall model-data fit, the pseudo 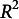 (see Section 3.1.4) based on the E2PL is 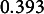 .

**Table 4:** RMSEs from the 10-fold cross-validation

Iteration	RMSE	
(*k*)	E2PL	GPCM
1	0.93	1.02
2	0.91	0.98
3	0.93	1.00
4	0.92	1.00
5	0.92	1.00
6	0.92	0.99
7	0.91	0.98
8	0.92	0.98
9	0.92	1.01
10	0.93	1.01
Overall	0.92	1.00

### 6.3 Discussion

It is notable from Figure 10 that the model-data fits of the two models appear visually comparable, making it difficult to judge the superiority of one model over the other based on visual inspection alone. This highlights the potential advantage of the E2PL in reducing the number of item parameters while maintaining an equivalent model-data fit, a conclusion supported by the 10-fold cross-validation results.

The cross-validation results imply that the better predictive performance of the E2PL may be due to the number of item parameters required by each model. The GPCM uses five item parameters per item, whereas the E2PL requires only three. This reduction in the number of parameters could have contributed to the E2PL’s superior performance, as the GPCM includes 188 additional parameters (2 parameters 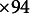 items) in this example.

The results support the application of the E2PL to sparse polytomous item response data. Although the results of this section may not be generalizable, the results show that the E2PL can be an alternative to polytomous IRT models when a parsimonious model is preferred, especially for sparse data. The rating scale model (Andrich, 1982) can be another option for model parsimony. However, the rating scale model assumes that its threshold parameters are equally spaced on the 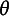 scale. For some situations, including the current example, the scale conversion of this section can be more justifiable than the rating scale model’s assumption.

## 7 Conclusion

This article proposed an IRT model that is suitable for dealing with continuous and sparse polytomous item response data. While existing models can provide useful psychometric insight, they often fall short in terms of model assumptions, interpretations, and parameter estimation to be used in areas, such as CAT and test linking/equating. The model presented in this study extends the widely-used 2PL model (Birnbaum, 1968) by adding an error term that follows a shifted beta distribution. The formulation of the model is well aligned with the generalized latent variable model framework (McCullagh & Nelder, 1989; Skrondal & Rabe-Hesketh, 2004), incorporating the same mean structure as the 2PL through the logit link function, along with an additional precision parameter to model the dispersion structure. Consequently, researchers and practitioners familiar with IRT models can easily apply and interpret this extended model. The model’s structural resemblance to the 2PL also makes the model preferable when a mixed-format test is constructed with continuous response items.

The mean–precision parameterization of the beta distribution allows the separation of the item discrimination and difficulty parameters from the precision parameter 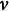 . This separation enables the discrimination and difficulty parameters to control the expected response 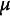 , while the precision parameter controls response variability conditional on 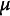 . Interestingly, the statistical dependence between 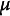 and 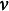 allows the model to yield communality and unique dispersion indices that reflect item information, with these indices remaining invariant under scale transformation. This characteristic parallels the practical distinction between FA and IRT, where item thresholds (i.e., item difficulty) play a more central role in IRT than in FA. Additionally, the beta distribution can model asymmetric or zero-one-inflated item response distributions.

Unless the precision parameter 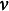 is close to or below 3, the information function of the model is a symmetric, bell-shaped curve peaking at 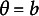 . This feature is especially useful in CAT for administering items that provide maximum information. Meanwhile, when the item discrimination and difficulty parameters are shared between the 2PL and the E2PL, the E2PL offers greater item information than the 2PL. This suggests that continuous item responses can yield more information than binary responses, as demonstrated in Section 5 with items providing substantial information over a wide 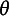 range.

However, because observed item responses are likely to be discrete in the strict sense (e.g., from 0% to 100% with the increment of 1%), the continuity assumption on item responses may overestimate item information to some extent. Future studies can investigate the effect of this assumption violation and apply continuity correction methods to appropriately adjust the amount of information. Additionally, especially in survey data collected using slider items, the impact of response biases toward extreme values can be more detrimental than with polytomous items.

Model parameters can be estimated using the MML-EM procedure (Bock & Aitkin, 1981), and the simulation study demonstrated robust parameter recovery and stable estimation even with sample sizes as small as 250. All MML-EM procedures converged successfully, and the computational time was within acceptable limits for practical applications.

The model’s application to sparse polytomous data was further tested via 10-fold cross-validation on empirical data, where it outperformed the GPCM (Muraki, 1992) in predictive accuracy. The improvement in performance can be attributed to the model’s fewer item parameters compared to the GPCM, which is advantageous when sample sizes are limited. However, the results from the empirical example may not guarantee a parallel effect when the model is applied to a different dataset. In addition, the application of the model to polytomous data entails a scale conversion of item responses. While the conversion can be justified in many cases, including the example of this article, it may not be justified for other cases. Nonetheless, the model proposed in this article can be a useful alternative to polytomous IRT models when observed responses per score categories are insufficient.

For greater parsimony, the model could be simplified by fixing the item discrimination parameter *a* across all items, thereby aligning the expected response 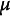 with the ICF of the Rasch model (Rasch, 1960). This simplification retains the core properties of the E2PL while reducing the number of item parameters. Additionally, future work could extend the E2PL to multidimensional applications by adopting a vectorized model equation.

The parameter estimation software developed for this article is publicly available in R via the IRTest_Cont function of the IRTest package (Li, 2025; R Core Team, 2024). Also, further application studies of the model may motivate item writers to develop and use more flexible item types, including continuous response items, when those items are expected to enhance the validity and reliability of assessments.

## A Appendix

### A.1 Item parameter estimation

In the MML-EM procedures, the iteration of the expectation step (E-step) and the maximization step (M-step) can be terminated when changes in parameter estimates drop below a predefined threshold.

#### A.1.1 Marginal log-likelihood

Let  denote the marginal log-likelihood,  be the latent distribution, and  be the *q*th quadrature point within the quadrature scheme of the MML-EM procedure (  ). Then, the marginal log-likelihood can be expressed as follows:

(A.1)

where the integration is approximated by the summation and  is a normalized density after discretization.

#### A.1.2 E-step of the MML-EM procedure

In the E-step of the MML-EM procedure, expected values are calculated. The quantity  implies the expected probability of the *j*th examinee’s ability parameter belonging to the *q*th grid in the latent space.

(A.2)

In addition,  indicates the expected population frequency at the *q*th grid.

#### A.1.3 M-step of the MML-EM procedure

In the M-step, the marginal log-likelihood becomes more tractable using  and Jensen’s inequality:

(A.3)

Since only the first term of the equation’s last line is dependent on the item parameters, the item parameters can be estimated by maximizing it. Moreover, by the local independence assumption, the item parameters can be estimated separately for each item.

**Newton–Raphson Method** The Newton–Raphson method can be used to find item parameter estimates that maximize the marginal log-likelihood of the M-step. Instead of  ,  is estimated for a more numerically feasible estimation, as  can take only positive values. For notational brevity, item indices are omitted and additional quantities are introduced:

(A.4)

and

(A.5)

In addition, as omitted responses are not counted by the index *j*, the total number of test takers may vary across items.

Then, the first derivatives of the item parameters are as follows:

(A.6)

(A.7)

and

(A.8)

where  is a digamma function.

The expectations of the second derivatives are as follows:

(A.9)

(A.10)

(A.11)

(A.12)

(A.13)

and

(A.14)

where  is a trigamma function.

On the *k*th iteration, the parameters are updated using the following equation:

(A.15)

The Newton–Raphson iteration is terminated when changes in parameter estimates drop below a predefined threshold.

### A.2 Ability parameter estimation

Assuming that test takers are independent, their ability parameter estimates can be estimated individually. Thus, the index *j* for the test takers is dropped for notational brevity and  indicates items. For the MLE, the likelihood to be maximized is simply the product of individual response probabilities:

(A.16)

For the WLE, the likelihood is the sum of  and a specific function to obtain unbiased estimate. The first derivative of the WLE likelihood will be directly provided.

#### A.2.1 EAP

The  can be regarded as a posterior distribution of the ability parameter. Therefore, EAP scores can be calculated as the posterior mean of the latent distribution.

(A.17)

#### A.2.2 MLE & WLE

Again, the Newton–Raphson method is introduced to calculate MLE and WLE. The Newton–Raphson procedure is terminated when changes in parameter estimates drop below a predefined threshold.

MLE The first and second derivatives are as follows:

(A.18)

and

(A.19)

On the *k*th iteration, MLE is updated using the following equation:

(A.20)

WLE While the second derivative is the same as in MLE, a weight is added to the first derivative of MLE:

(A.21)

and

(A.22)

Likewise, on the *k*th iteration, WLE is updated using the following equation:

(A.23)
